# Low temperature dehydrogenation properties of ammonia borane within carbon nanotube arrays: a synergistic effect of nanoconfinement and alane†

**DOI:** 10.1039/d0ra02283g

**Published:** 2020-05-20

**Authors:** Zhijie Cao, Liuzhang Ouyang, Michael Felderhoff, Min Zhu

**Affiliations:** School of Materials Science and Engineering, Key Laboratory of Advanced Energy Storage Materials of Guangdong Province, South China University of Technology Guangzhou 510641 PR China meouyang@scut.edu.cn; Advanced Energy Storage Materials and Devices Laboratory, School of Physics and Electronic-Electrical Engineering, Ningxia University Yinchuan 750021 PR China; Max-Planck-Institut für Kohlenforschung Kaiser-Wilhelm-Platz 1 45470 Mülheim Germany felderhoff@mpi-muelheim.mpg.de

## Abstract

Ammonia borane (AB, NH_3_BH_3_) is considered as one of the most promising hydrogen storage materials for proton exchange membrane fuel cells due to its high theoretical hydrogen capacity under moderate temperatures. Unfortunately, its on-board application is hampered by the sluggish kinetics, volatile byproducts and harsh conditions for reversibility. In this work, AB and AlH_3_ were simultaneously infiltrated into a carbon nanotube array (CMK-5) to combine the synergistic effect of alane with nanoconfinement for improving the dehydrogenation properties of AB. Results showed that the transformation from AB to DADB started at room temperature, which promoted AB to release 9.4 wt% H_2_ within 10 min at a low temperature of 95 °C. Moreover, the entire suppression of all harmful byproducts was observed.

## Abstract 2

Ammonia borane (AB, NH_3_BH_3_) is considered as one of the most promising hydrogen storage materials for proton exchange membrane fuel cells due to its high theoretical hydrogen capacity under moderate temperatures.

## Graphical abstract

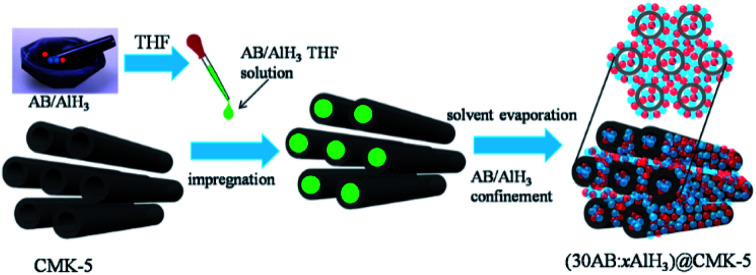

## 1. Introduction

Large-scale applications of hydrogen in proton exchange membrane fuel cells (PEMFCs) are greatly promoted by the booming developments of efficient hydrogen storage technology.^1^ Hydrides composed of light elements are an option due to their high volumetric storage capacities and compact nature of hydrogen storage.^2,3^ AB is a promising light weight hydride because of its remarkable hydrogen capacity (19.6 wt% and 140 g L^−1^), moderate desorption temperatures and relatively high air stability.^4–6^ Upon thermal decomposition, the liberation of H_2_ from AB takes place in three steps (eqn (1)–(3)):^7–9^

1 *n*NH_3_BH_3_ → (NH_2_BH_2_)_*n*_ + *n*H_2_ (90–120 °C, 6.5 wt%)

2 (NH_2_BH_2_)_*n*_ → (NHBH)_*n*_ + *n*H_2_ (120–200 °C, 6.9 wt%)

3 (NHBH)_*n*_ → *n*BN + *n*H_2_ (>500 °C, 7.3 wt%)

Owing to the extreme high desorption temperature of step (3) (>500 °C), only the hydrogen release from eqn (1) and (2) below 200 °C are regarded as possible for practical applications in combination with fuel cell systems. For PEMFCs, however, there are still three main challenges involved in the first two steps: (a) the emission of detrimental byproducts (*i.e.*, ammonia, diborane, and borazine); (b) relatively high desorption temperature (100–200 °C); (c) poor reversibility. To overcome these obstacles, several strategies have been explored, including the infiltration of AB in porous structures (nanoconfinement),^10–12^ the addition of metal based catalysts,^13–15^ modifying thermodynamics through chemical alteration,^16–18^*etc.* Among them, nanoconfinement is an effective approach to positively affect the dehydrogenation thermodynamics and kinetics of AB, which originates from the effects of increased surface energy, induced defects and vacancies as well as shortened diffusion distances.^19^ The pioneering work by Gutowska *et al.* demonstrated that the decrease of desorption temperature and the suppression of borazine were simultaneously achieved by the capsulation of AB into mesoporous silica.^10^ However, they didn't mention the problem of NH_3_ liberation during the decomposition process, which would severely poison PEMFCs even at a very low level (1 ppm).^20^ This issue could be solved by the combination of nanostructure confinement with metal catalysts. Li *et al.* incorporated AB into a lithium (Li) functionalized ordered mesoporous carbon framework (CMK-3), and considerable enhancement of dehydrogenation properties as well as complete suppression of all volatile byproducts could be observed in the AB/Li–CMK-3 composite.^21^ Since then, various surface functionalized nanoscaffolds have been explored, including carbon nanotubes,^22–24^ metal–organic frameworks,^25–27^ mesoporous 3D boron nitride,^28^ Pd/halloysite nanotubes,^29^*etc.* On the other hand, the destabilization of AB and suppression of volatile byproducts could also be achieved by compositing with metal hydrides (MH).^30–33^ Kang *et al.* found that the mechanically milled mixture of AB and MgH_2_ could release 8 wt% H_2_ within 4 h at around 100 °C without the byproducts of diborane and borazine.^31^ In AB-MH systems, Nakagawa *et al.* demonstrated that Pauling electronegativity of M is good to indicate the amount of undesired byproducts (NH_3_ and B_2_H_6_), and the level of NH_3_ decreased with the increase of electronegativity.^32^ Therefore, AlH_3_ is more effective than MgH_2_ on inhibiting the emission of NH_3_ due to the higher electronegativity of Al. Compared with nanoconfined systems, the dehydrogenation temperatures of these MH-doped composites are relatively high (>100 °C).

These observations and advancements imply that further improvements of the dehydrogenation properties of AB can be expected through the combination of nanoconfinement and MH. CMK-5, an ordered mesoporous carbon material possessing very high specific surface area (up to 2000 m^2^ g^−1^) and bimodal porosity^34^ (much higher than that of SBA-15 (ref. 35) and CMK-3 (ref. 36)), is a perfect host material for fabricating of nanoconfined materials.^37–39^ However, to our knowledge, CMK-5 has never been used as nanoscaffold for hydrogen storage materials before. Therefore, the composites of AB and AlH_3_ were encapsulated into CMK-5 in this work, and the possible improved mechanism of dehydrogenation properties was proposed.

## 2. Experimental section

### Materials synthesis

**Fig. 1:**
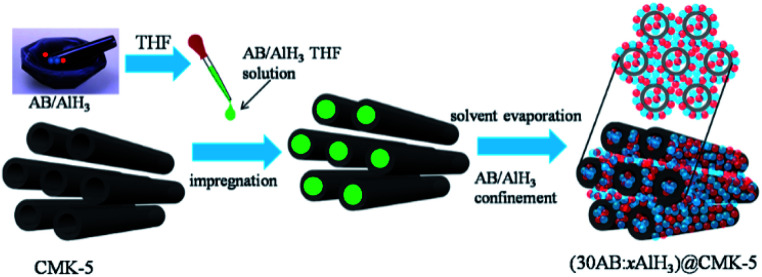
The schematic illustration of the preparation process of (30AB : *x*AlH_3_)@CMK-5 (blue balls: AB, red balls: AlH_3_).

CMK-5 was prepared according to the literature.^38^ Alane was synthesized by the organometallic method.^40,41^ Ammonia borane (97 wt%) purchased from Sigma-Aldrich was used directly without further purification. Before use, CMK-5 was degassed for 20 h at 160 °C to remove adsorbed moisture and gases. Composites of *x* wt% AB (*x* = 20, 30, 40, 50) and CMK-5 with different percentages of AB in CMK-5 were denoted as *x*AB@CMK-5, and composites of 30 wt% AB and *x* wt% AlH_3_ (*x* = 10, 15, 30) in CMK-5 were denoted as (30AB : *x*AlH_3_)@CMK-5. Schematic illustration of the preparation process of (30AB : *x*AlH_3_)@CMK-5 is shown in Fig. 1. AB and AlH_3_ was physically mixed in a mortar by hand-milling and then dissolved in anhydrous THF at room temperature. Afterwards, the THF solution of AB and AlH_3_ was instilled into CMK-5, and the resulted suspension was further stirred for 2 h at room temperature. Finally, the solvent was evaporated under vacuum overnight. A physical mixture of same amount of AB and AlH_3_ (denoted as AB/AlH_3_) was also prepared for comparison. All preparation procedures were carried out under purified argon atmosphere in a glove box or using standard Schlenk technique.

### Characterization

Wide angle X-ray diffraction (XRD) patterns were recorded on a STOE STADI P diffractometer in Debye–Scherrer transmission geometry with Cu/Kα radiation, and all samples were sealed into glass capillaries with a diameter of 0.5 mm for measurements. Small angle XRD patterns were collected on a Stoe *θ*–*θ* diffractometer in Bragg–Brentano geometry with Cu/Kα radiation. The nitrogen adsorption–desorption curves were measured on a NOVA 4200e instrument at −196 °C. All these samples were degassed under vacuum for 24 h at room temperature before measurements. The BET surface areas were obtained from the data in the relative pressure range of 0.05 and 0.20. The pore volumes and pore size distributions were calculated from the desorption branches of isotherms by employing the Barrett–Joyner–Halenda (BJH) model. High-resolution scanning electron microscope (HRSEM) images, and transmission electron microscope (TEM) images were operated on a Hitachi S-5500 ultrahigh-resolution cold field emission scanning microscope. Thermogravimetric analysis (TGA) and differential scanning calorimeter (DSC) measurements were performed on a TGA/DSC STAR^e^ system with a heating rate of 5 °C min^−1^ under Ar atmosphere. The released gas was analyzed using mass spectrometry (MS, HPR-20 QMS) with an argon purge rate of 50 mL min^−1^. IR-spectra were measured with a Fourier transform infrared spectroscopy (FT-IR, Nicolet 560). Solid-state ^27^Al nuclear magnetic resonance (NMR) spectroscopy was recorded on a Bruker Avance 500WB spectrometer, using a Doty CP-MAS probe with no probe background. X-ray photoelectron spectroscopy (XPS) measurements were conducted on a Kratos HSI spectrometer with a hemispherical analyzer. The monochromatized Al K_α_ X-ray source (*E* = 1486.6 eV) was conducted at 15 kV and 15 mA. An analyzer pass energy of 40 eV was adopted for the narrow scans. The hybrid mode was employed as lens mode. The base pressure in analysis chamber was 4 × 10^−7^ Pa during the process. The values of binding energy were referred to the C 1s peak (284.5 eV) with the purpose of accounting for charging effects.

## 3. Results and discussion

**Fig. 2:**
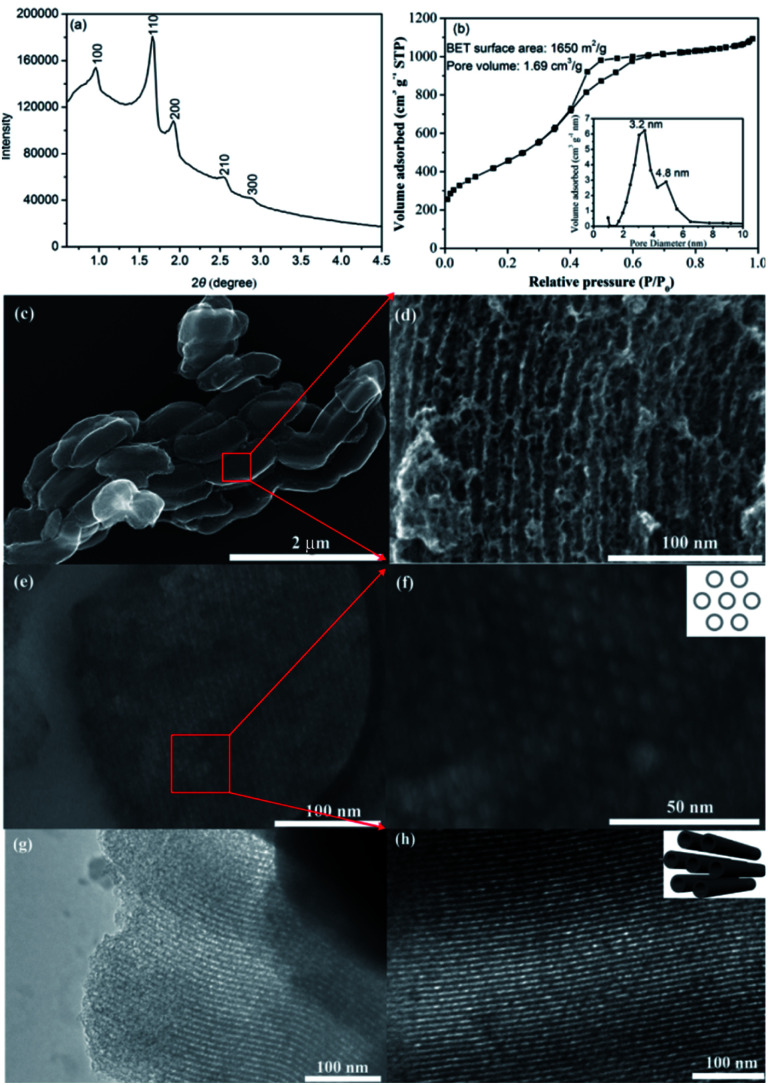
The properties of mesoporous CMK-5: (a) small angle XRD pattern, (b) N_2_ adsorption–desorption isotherm, inset is the pore size distribution, (c) SEM image, (d) magnified images of the red zone in (c), (e) cross section SEM image, (f) magnified image of the red zone in (e), (g and h) TEM images.

The structure of as-prepared CMK-5 was characterized by small angle XRD, N_2_ adsorption–desorption isotherm, SEM and TEM, and the results are presented in Fig. 2. Small angle XRD measurement (Fig. 2a) gives peaks of (100), (110), (200), (210) and (300) reflections, indicating the ordered 2D hexagonal structure of CMK-5.^35^ N_2_ adsorption–desorption analysis (Fig. 2b) shows that the BET surface area and pore volume of CMK-5 are 1650 m^2^ g^−1^ and 1.69 cm^3^ g^−1^, respectively. The typical structure of CMK-5 with bimodal porosity is proved by the two maxima of pore size distribution (inset image of Fig. 2b). SEM images (Fig. 2c and d) reveal the rod-like morphology of CMK-5, and further SEM images of cross section (Fig. 2e and f) as well as TEM images (Fig. 2g and h) confirm its uniform and ordered 2D hexagonal structure.

**Fig. 3:**
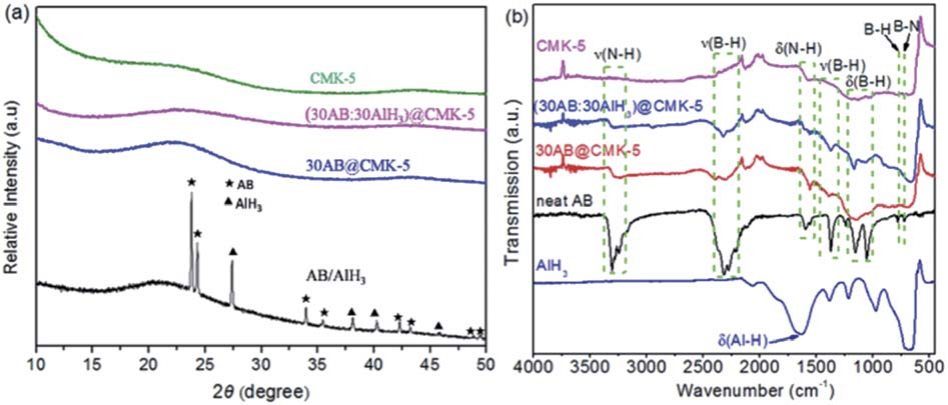
(a) XRD and (b) FT-IR patterns of CMK-5, 30AB@CMK-5, and AB/AlH_3_ (30AB : 30AlH_3_)@CMK-5.

**Fig. 4:**
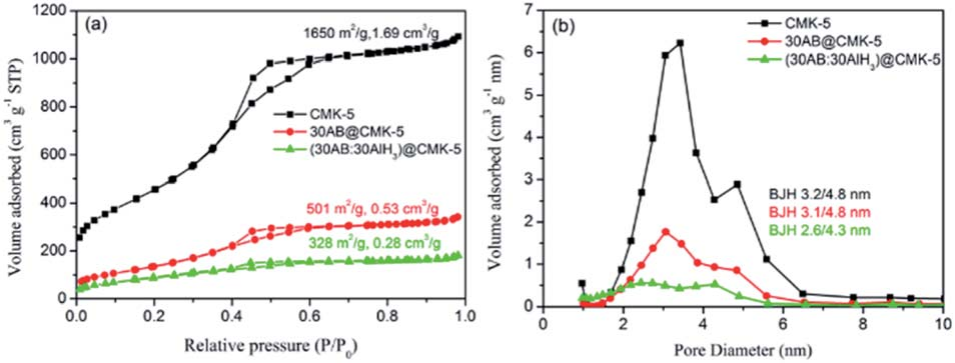
(a) N_2_ adsorption–desorption isotherms at −196 °C and (b) the pore size distributions of CMK-5, 30AB@CMK-5 and (30AB : 30AlH_3_)@CMK-5.

AB and/or AlH_3_ were loaded into CMK-5 by the infuse method, and the impregnation of them were confirmed by the XRD, FT-IR and N_2_ adsorption–desorption analysis. Fig. 3a shows the XRD patterns of CMK-5, 30AB@CMK-5, AB/AlH_3_ and (30AB : 30AlH_3_)@CMK-5. Compared with the obvious diffraction peaks of AB and AlH_3_ in the AB/AlH_3_ composite, these peaks become hardly observable after encapsulating into CMK-5 frameworks, indicating the dispersion of AB and AlH_3_ into mesoporous CMK-5 at a nano or amorphous state. As shown in Fig. 3b, the presence of N–H, B–H and weak Al–H stretching peaks in the FT-IR spectrum confirms the existence of AB and/or AlH_3_ in CMK-5. Fig. 4a and b show the N_2_ adsorption–desorption isotherms and pore size distributions of CMK-5, 30AB@CMK-5 and (30AB : 30AlH_3_)@CMK-5, respectively. Compared with pure CMK-5, the specific surface areas and pore volumes are declined dramatically from 1650 to 501 and 328 m^2^ g^−1^, and from 1.69 to 0.53 and 0.28 m^3^ g^−1^, respectively, for 30AB@CMK-5 and (30AB : 30AlH_3_)@CMK-5 (Fig. 4a). The corresponding pore size distributions also show a gradual loss of two maxima (Fig. 4b), further confirming that most of the pores of CMK-5 are filled or blocked by AB and/or AlH_3_.

**Fig. 5:**
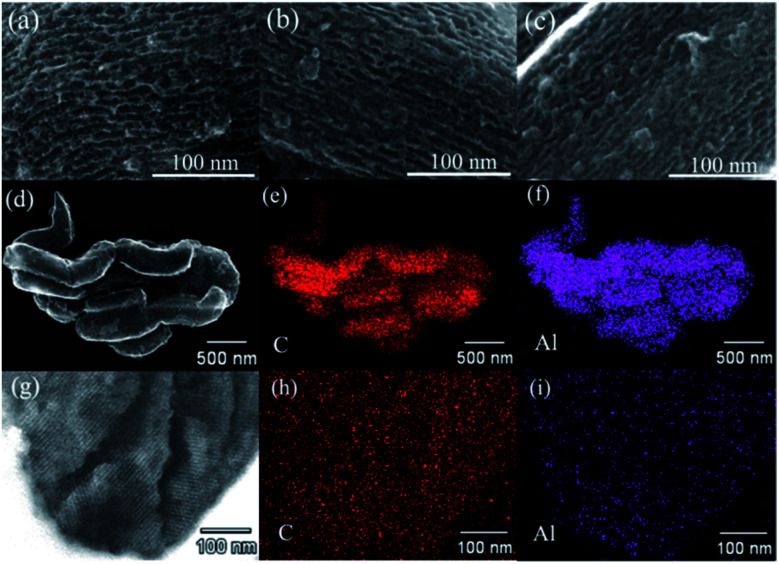
SEM images: (a) CMK-5, (b) 30AB@CMK-5, (c) (30AB : 30AlH_3_)@CMK-5; (30AB : 30AlH_3_)@CMK-5: (d) surface SEM image, and the corresponding EDS maps of (e) C and (f) Al, (g) cross section SEM image, and the corresponding EDS maps of (h) C and (i) Al.

**Fig. 6:**
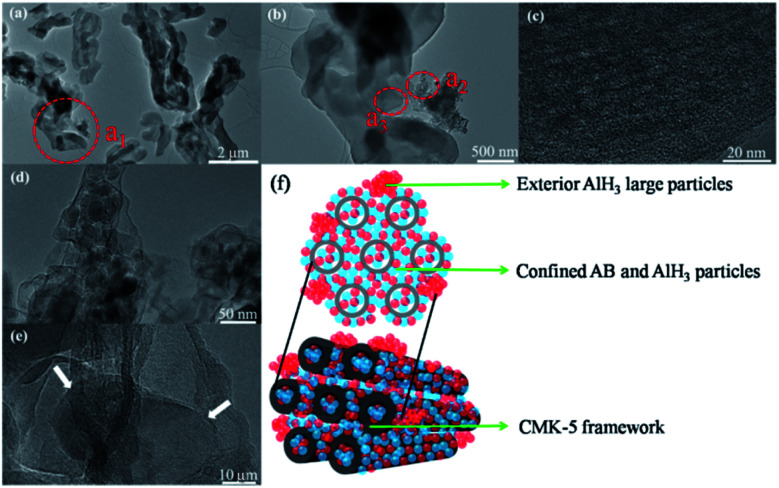
(30AB : 30AlH_3_)@CMK-5: (a) TEM image, (b) high-magnification TEM micrographs of the area delineated by dotted circle a_1_ in (a), (c) high-resolution TEM micrographs of the area delineated by dotted circle a_3_ in (b), (d and e) high-magnification TEM micrographs of the area delineated by dotted circle a_2_ in (b), (f) the schematic diagram of the actual structure.

To further assess the distribution of AB and/or AlH_3_, these confined samples are also characterized by SEM, and the results are shown in Fig. 5. In the pristine CMK-5 (Fig. 5a), the typical pore channels can be observed on the surface, while most of these pore channels in 30AB@CMK-5 (Fig. 5b) are occupied after the impregnation of AB. For (30AB : 30AlH_3_)@CMK-5 (Fig. 5c), no obvious channel feature of CMK-5 can be identified due to the accumulation of AB and AlH_3_. Further energy dispersive X-ray spectroscopy (EDS) mapping results of the surface (Fig. 5d–f) and cross section (Fig. 5g–i) of (30AB : 30AlH_3_)@CMK-5 indicate the well distribution of AlH_3_ both in and outside the channels of CMK-5. However, we failed to track the distribution of B and N through EDS due to their light nature and relatively low loaded amount here. In fact, AB was supposed to be well distributed in the inner and external surfaces of CMK-5 through the impregnation method.^21,23,40^ To further observe the distributions of AB and AlH_3_ in (30AB : 30AlH_3_)@CMK-5, TEM was also conducted and the results are presented in Fig. 6. As shown in Fig. 6a, (30AB : 30AlH_3_)@CMK-5 generally displays a rather homogeneous structure. An enlargement of the dotted circle a_1_ shows that there are some aggregated particles outside the pore channels (Fig. 6b), which was confirmed to be AlH_3_ by EDS result. Further enlargements of the dotted circle a_2_ (Fig. 6d and e) show that these AlH_3_ particles with a size of 20–40 nm stay in a highly crystalline state. While from the high-resolution TEM image of the dotted circle a_3_ (Fig. 6c), no obvious AB or AlH_3_ particles can be distinguished from the frameworks of CMK-5, indicating the possible amorphous structure of AB and AlH_3_. In combination with the SEM and TEM results, the schematic diagram of the actual structure of (30AB : 30AlH_3_)@CMK-5 is shown in Fig. 6f. The majority of AB and AlH_3_ uniformly distribute both in and outside the channels of CMK-5, while a small part of AlH_3_ gather to form large particles on the surface.

**Fig. 7:**
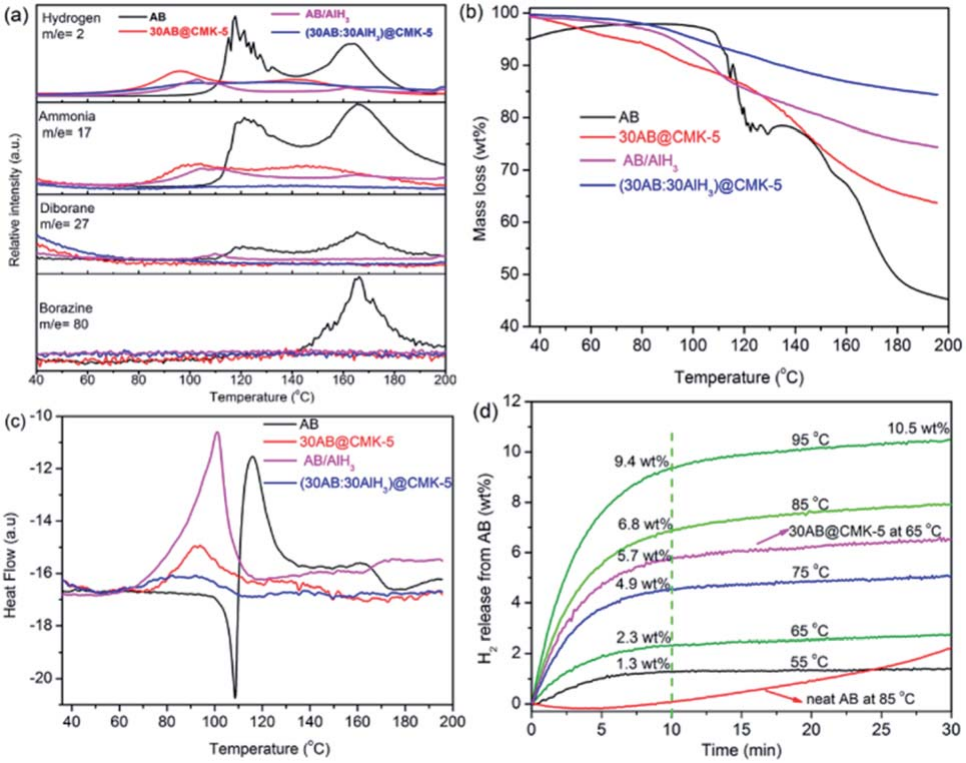
(a) MS, (b) TGA and (c) DSC curves of AB, 30AB@CMK-5, AB/AlH_3_ and (30AB : 30AlH_3_)@CMK-5 with a heating rate of 5 °C min^−1^, (d) isothermal desorption curves of (30AB : 30AlH_3_)@CMK-5.

MS, TGA, DSC and isothermal measurements were conducted to compare the dehydrogenation behaviors of AB, 30AB@CMK-5, AB/AlH_3_ and (30AB : 30AlH_3_)@CMK-5 (Fig. 7). All these data have been normalized to the loaded percentage of AB. As shown in Fig. 7a, the decomposition of neat AB displays a two-step process at 117 °C and 162 °C to release the first and second equivalent of hydrogen. Meanwhile, several byproducts (*i.e.*, ammonia, diborane and borazine) are detected in accompany with the liberation of hydrogen above 100 °C. The confinement of AB into CMK-5 contributes to considerable enhancements in the dehydrogenation behavior with an onset dehydrogenation temperature as low as 50 °C and two decomposition peaks at 98 °C and 140 °C. Moreover, a significant decrease of ammonia and a complete suppression of diborane and borazine are observed. These improvements are correlated with the nanoconfinement effect of CMK-5, which affects the decomposition thermodynamics and kinetics of AB.^41^ Notable reductions of dehydrogenation temperature and byproducts are also observable in AB/AlH_3_. However, the liberation of NH_3_ is still detectable during the heating process of 30AB@CMK-5 and AB/AlH_3_. Further inhibition of NH_3_ can be realized through the synergistic effect of CMK-5 and AlH_3_. As shown in Fig. S1,† the level of NH_3_ decreases with increasing the amount of AlH_3_ in (30AB : *x*AlH_3_)@CMK-5 (*x* = 10, 15, 30). A complete suppression of NH_3_ can be achieved by loading the same amount (30 wt%) of AB and AlH_3_ into CMK-5. As shown in Fig. 7b, the weight loss of 30AB@CMK-5, AB/AlH_3_ and (30AB : 30AlH_3_)@CMK-5 are obviously lower than neat AB, further indicating the suppression of byproducts. Because of partly liberated NH_3_, the weight loss from 30AB@CMK-5 is much higher than that of (30AB : 30AlH_3_)@CMK-5 under the same conditions. Fig. 7c shows the DSC curves of these samples. During the heating process, pure AB has a melting point at 108 °C before its decomposition, while this endothermic peak cannot be detected in AB@CMK-5, AB/AlH_3_ and (30AB : 30AlH_3_)@CMK-5. This indicates that the first equivalent H_2_ is released prior to the melting of AB in these samples.^42^ On the basis of integral calculation from DSC curves, the decomposition reaction enthalpies of neat AB and AB/AlH_3_ were calculated to be −19.9 and −19.7 kJ mol^−1^, respectively, which is in agreement with reported values (−21 kJ mol^−1^) for pure AB.^10,21,43^ The decomposition reaction enthalpies of AB in 30AB@CMK-5 and (30AB : 30AlH_3_)@CMK-5 increase to −2.9 and −2.0 kJ mol^−1^, respectively, due to the destabilization effect of nanoconfinement. Fig. 7d shows the isothermal dehydrogenation curves of (30AB : 30AlH_3_)@CMK-5 at different temperatures. For comparison, the dehydrogenation curves of neat AB at 85 °C and 30AB@CMK-5 at 65 °C were also presented. Even at low temperatures of 65 °C and 55 °C, AB in (30AB : 30AlH_3_)@CMK-5 can liberate 2.3 wt% and 1.2 wt% H_2_ within 10 min. Increasing temperature to 85 °C, a capacity of 6.8 wt% H_2_ can be released in 10 min while only a very small amount of hydrogen (0.2 wt%) is detected from neat AB within the same time. At temperature up to 95 °C, the amount of released hydrogen in 10 min increases to 9.4 wt% and reaches 10.5 wt% in 30 min. These results indicate a kinetic improvement of the decomposition process of AB through the synergistic effect of nanoconfinement and alane.

**Fig. 8:**
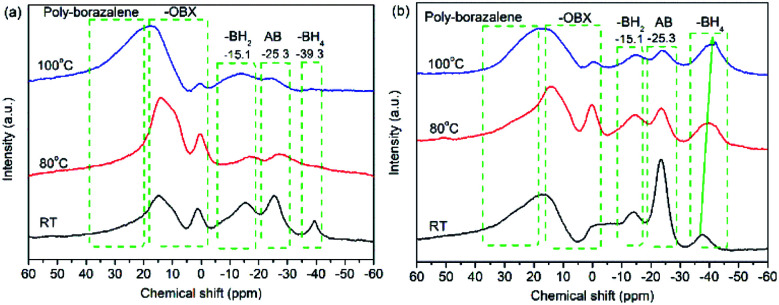
Solid-state ^11^B NMR spectrum of the composites: (a) 30AB@CMK-5, (b) (30AB : 30AlH_3_)@CMK-5.

**Fig. 9:**
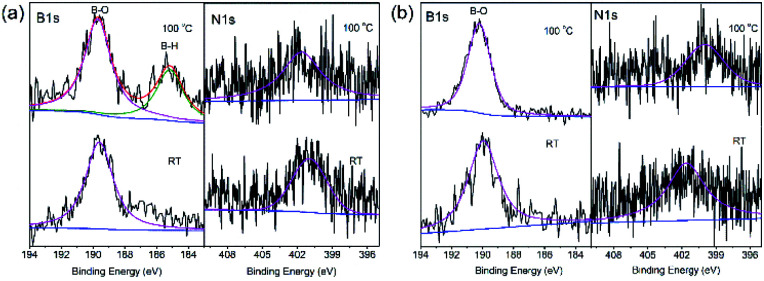
B 1s and N 1s XPS spectra of (a) 30AB@CMK-5, (b) (30AB : 30AlH_3_)@CMK-5 at different temperatures.

To understand the decomposition mechanism, solid-state ^11^B NMR and XPS spectra of 30AB@CMK-5 and (30AB : 30AlH_3_)@CMK-5 before and after heating to 80 °C and 100 °C were recorded, and the results are presented in Fig. 8 and 9. As shown in Fig. 8a, the spectrum of 30AB@CMK-5 at room temperature shows one resonance peak at −25.3 ppm for unreacted AB, and two additional peaks at −15.1 and −39.3 ppm for BH_2_ and BH_4_ of the diammoniate of diborane (DADB), [(NH_3_)_2_BH_2_^+^][BH_4_^−^]. DADB is a reactive product, the production of which is a crucial nucleation event that can trigger the rapid release of hydrogen, but its formation in pure AB required a heating period of 30 min at 85 °C.^44^ In our case, the transformation from AB to DADB starts in 30AB@CMK-5 during the impregnation or drying process at room temperature, demonstrating the destabilization effect of nanoconfinement on AB. Usually nanoscaffolds containing reactive oxygen functional groups can react with AB during the heating process.^10,21,45^ XPS results also show hydroxyl groups in CMK-5 (Fig. S2†). Hence, the formation of organic borates (–OBX) at 1.1 and 14.9 ppm could be the result of the reaction between AB and surface hydroxyl groups,^46^ resulting in the cleavage of B–N and B–H bonds of AB to release NH_3_ and H_2_ during the decomposition process.^21^ After heating up to 80 °C, the resonances of AB and DADB at −15.1, −25.3 and −39.3 ppm obviously broaden, and the intensity significantly decreases. After increasing the temperature to 100 °C, these two starting materials are almost consumed and disappeared. A broad peak appears at about 20 ppm, which is a result of the formation of polyaminoborane (PAB) species after the release of one equivalent of H_2_ from AB. No borazine compound can be observed at 31.1 ppm.^47^ For (30AB : 30AlH_3_)@CMK-5, as shown in Fig. 8b, the resonance peaks at room temperature are similar to 30AB@CMK-5, while the spectra at 80 °C and 100 °C are quite different. After heating from room temperature to 100 °C, the peak intensity of AB gradually decreases while that of DADB stays almost unchanged with only a slight shift of the BH_4_ position from −37.6 ppm to −40.2 ppm, suggesting that the addition of AlH_3_ polymerizes DADB. Fig. 9 shows the B 1s and N 1s XPS spectra of 30AB@CMK-5 and (30AB : 30AlH_3_)@CMK-5 at different temperatures. The peaks at ∼189.5 eV correspond to B–O bond, which is close to a structure with oxidized trigonal geometry (BC_2_O, 190.0 eV),^48^ further confirming the reaction between AB and surface hydroxyl groups. After thermal decomposition at 100 °C, the peak associated with B–H bond appears at ∼185.2 eV in 30AB@CMK-5 (Fig. 9a) while not in (30AB : 30AlH_3_)@CMK-5 (Fig. 9b). Besides, there is an increase in the intensity of B–O bond both in 30AB@CMK-5 and (30AB : 30AlH_3_)@CMK-5. From the XPS elemental composition analysis, as shown in Table 1, obvious reduction of N content from 3.13 to 1.44 wt% is detected in 30AB@CMK-5 after heat treatment due to the release of NH_3_. While there is almost no change of the N content in (30AB : 30AlH_3_)@CMK-5 under the same condition, this demonstrates the immobilization of N in this composite. These results indicate that, on the one hand, AB reacts with surface located functional groups to form organic borates (–OBX), and further addition of AlH_3_ would change the decomposition route of confined AB in (30AB : 30AlH_3_)@CMK-5 because of coulombic attraction between the hydridic H^*δ*−^ of AlH_3_ and protonic H^*δ*+^ of NH_3_ moieties in AB.^32,49^ On the other hand, the hydridic H^*δ*−^ of AlH_3_ and protonic H^*δ*+^ of NH_3_ moieties in DADB also results in the polymerization of DADB as –NH_2_BH_2_NH_2_BH_4_–. The interaction between Al–H and N–H can be further confirmed by the FT-IR spectrometry in Fig. S5.† The stretching and bending modes of the N–H and B–H bonds of AB can be clearly identified in both 30AB@CMK-5 and (30AB : 30AlH_3_)@CMK-5. As shown in Fig. S5a,† the intensity of N–H, B–H and B–N bonds in 30AB@CMK-5 decrease after the thermal reaction, implying the decomposition of AB and DADB. For (30AB : 30AlH_3_)@CMK-5, the intensity of N–H bonds also decline whereas the stretching bonds of B–N are almost constant with only a slight shift of the positions (marked by arrows in Fig. S5b†). This indicates that DADB undergo a structural modification instead of thermal decomposition due to the addition of AlH_3_. These results are consistent with the NMR results above.

**Table 1:** Elemental composition of 30AB@CMK-5 and (30AB : 30AlH_3_)@CMK-5 from XPS analysis

Sample	*T,* °C	B 1s, wt%	C 1s, wt%	N 1s, wt%	O 1s, wt%	Others
30AB@CMK-5	RT	3.59	62.07	3.13	16.45	14.77
	100	7.32	34.90	1.44	29.00	27.33
(30AB : 30AlH_3_)@CMK-5	RT	6.42	63.77	2.95	17.94	8.92
	100	5.01	62.19	2.89	14.71	15.20

**Fig. 10:**
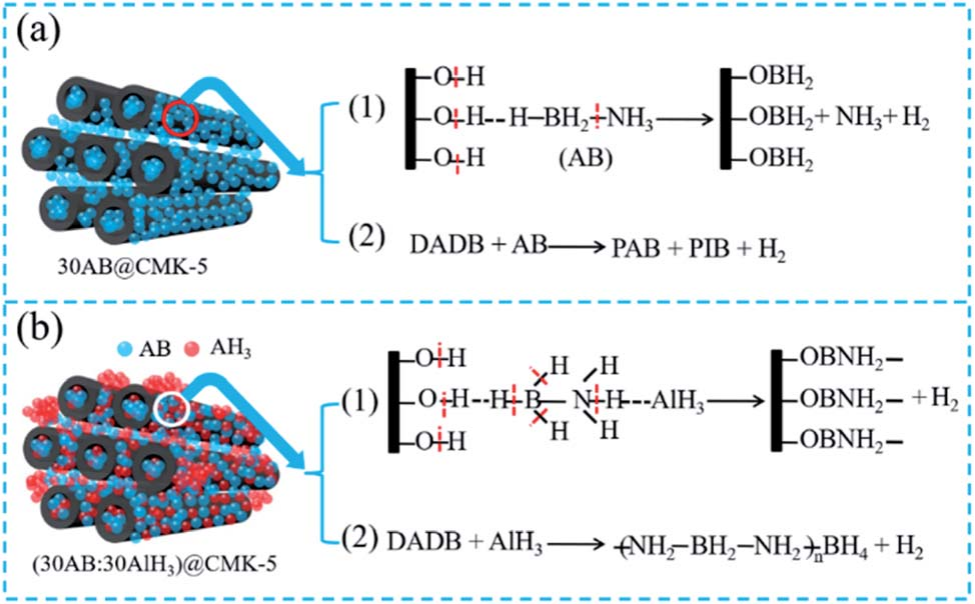
The proposed decomposition mechanism of (a) 30AB@CMK-5 and (b) (30AB : 30AlH_3_)@CMK-5.

According to the abovementioned analysis, the decomposition pathways of 30AB@CMK-5 and (30AB : 30AlH_3_)@CMK-5 can be illustrated in Fig. 10. As shown in Fig. 10a, the decomposition of 30AB@CMK-5 can release hydrogen in two different ways: (1) AB particles on close contact with the surface of CMK-5 decompose *via* the reaction between –BH_3_ and –OH to generate –OBH_2_ species, resulting in the cleavage of B–N and B–H bonds to liberate H_2_ and NH_3_; (2) AB molecules inside CMK-5 channels would release H_2_ through the pathway from DADB to PAB, PIB and H_2_ because of the nanoconfinement effect of CMK-5. As shown in Fig. 10b, the attraction between AlH_3_ and NH_3_ in (30AB : 30AlH_3_)@CMK-5 could influence these two decomposition steps as follows: (1) coulombic attraction between the hydridic H^*δ*−^ of AlH_3_ and the protonic H^*δ*+^ of NH_3_ moieties in AB would result in the cleavage of B–H and N–H bonds to prompt the production of –OBNH_2_– like structures, which is responsible for the immobilization of N and thus the suppression of NH_3_; (2) hydridic H^*δ*−^ of AlH_3_ reacts with active DADB to polymerize as –NH_2_BH_2_NH_2_BH_4_– species, leading to the stabilization of BH_4_ during the heating process.

## 4. Conclusions

In summary, a new AB and AlH_3_ nanostructure nanoconfined composite, (30AB : 30AlH_3_)@CMK-5, has been fabricated for the possible applications in combination with low temperature PEMFCs. Results demonstrated that the synergetic effect of nanoconfinement of CMK-5 and AlH_3_ in (30AB : 30AlH_3_)@CMK-5 dramatically improve the dehydrogenation thermodynamics and kinetics of AB, meanwhile suppress the liberation of byproducts. AB in this framework composite can release 9.4 wt% H_2_ within 10 min at a low temperature of 95 °C, and the dehydrogenation enthalpy increases to −2.0 kJ mol^−1^ from the pristine value of −19.7 kJ mol^−1^. This advancement demonstrates that the combination of nanoconfinement with MH could be further explored to improve the hydrogen storage properties of AB and/or other hydride systems.

## Conflicts of interest

There are no conflicts to declare.

## Supplementary Material

RA-010-D0RA02283G-s001
