# Supplementary material for: Low temperature dehydrogenation properties of ammonia borane within carbon nanotube arrays: a synergistic effect of nanoconfinement and alane
Source: RSC Adv. 2020 May 20;10(32):19027–33. doi: 10.1039/d0ra02283g (PMC9053937; doi:10.1039/d0ra02283g)
Supplement: RA-010-D0RA02283G-s001 [file RA-010-D0RA02283G-s001.pdf]

Electronic Supplementary Material (ESI) for RSC Advances.
This journal is © The Royal Society of Chemistry 2020

Electronic Supplementary Information

Low temperature dehydrogenation properties of ammonia borane within carbon nanotube arrays: A synergistic effect of nanoconfinement and alane

Zhijie Cao[a,b,c], Liuzhang Ouyang[a]*, Michael Felderhoff[c]*, Min Zhu[a]

[a]School of Materials Science and Engineering, Key Laboratory of Advanced Energy Storage Materials of Guangdong Province, South China University of Technology, Guangzhou, 510641, PR China

[b]Advanced Energy Storage Materials and Devices Laboratory, School of Physics and Electronic-Electrical Engineering, Ningxia University, Yinchuan, 750021, PR China

[c]Max-Planck-Institut für Kohlenforschung, Kaiser-Wilhelm-Platz 1, 45470 Mülheim, Germany

*Corresponding author: Liuzhang Ouyang, E-mail: meouyang@scut.edu.cn; Tel.: 86-20-87114253. Michael Felderhoff, E-mail: felderhoff@mpi-muelheim.mpg.de. Tel.: 49(0)2083062368.

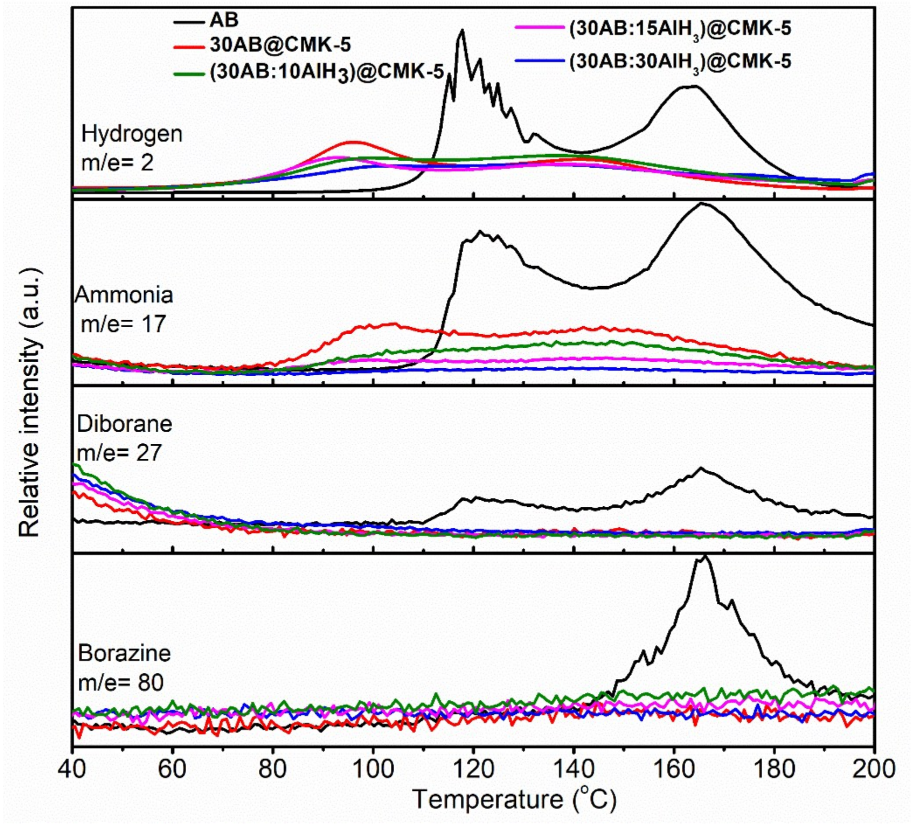

Figure S1. MS patterns of (30AB:xAlH$_3$)@CMK-5 (x = 10, 15, 30).

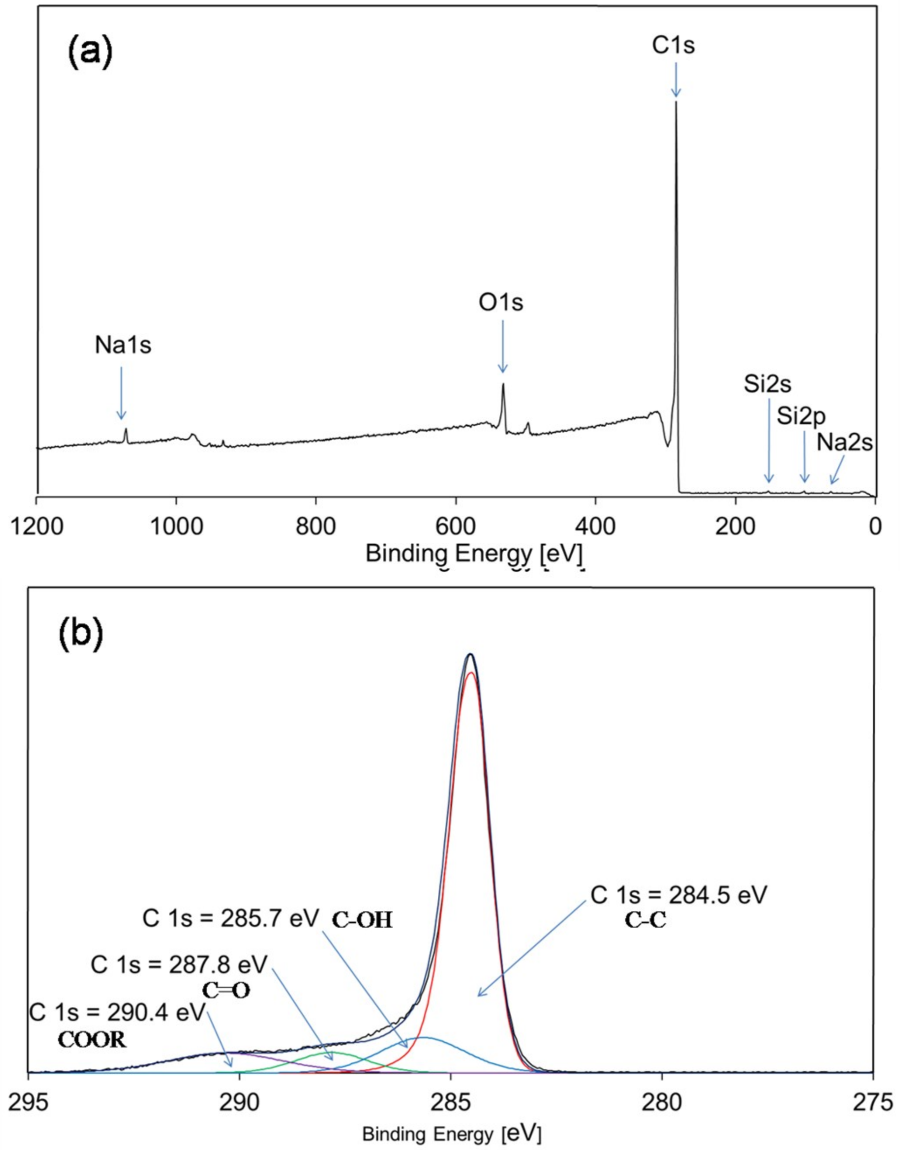

Figure S2.(a) XPS and (b) C1s XPS spectra for CMK-5.

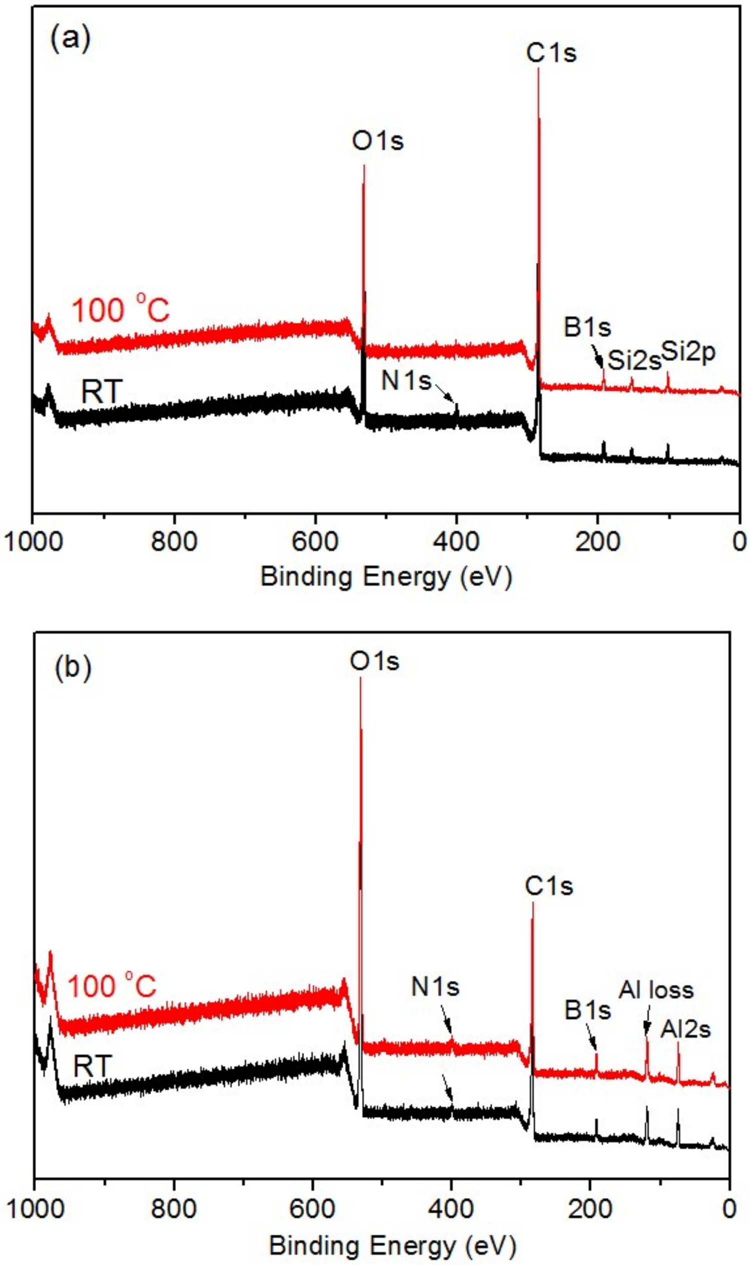

Figure S3. XPS spectra of (a) 30AB@CMK-5 and (b) (30AB:30AlH_3)@CMK-5 at different temperatures.

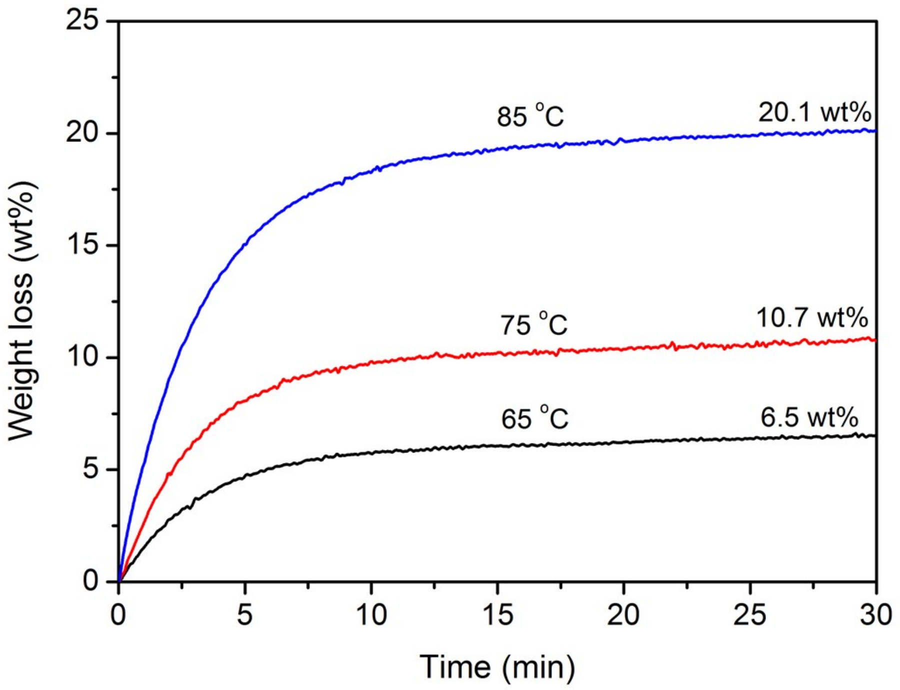

Figure S4. Isothermal dehydrogenation of 30AB@CMK-5 at different temperatures.

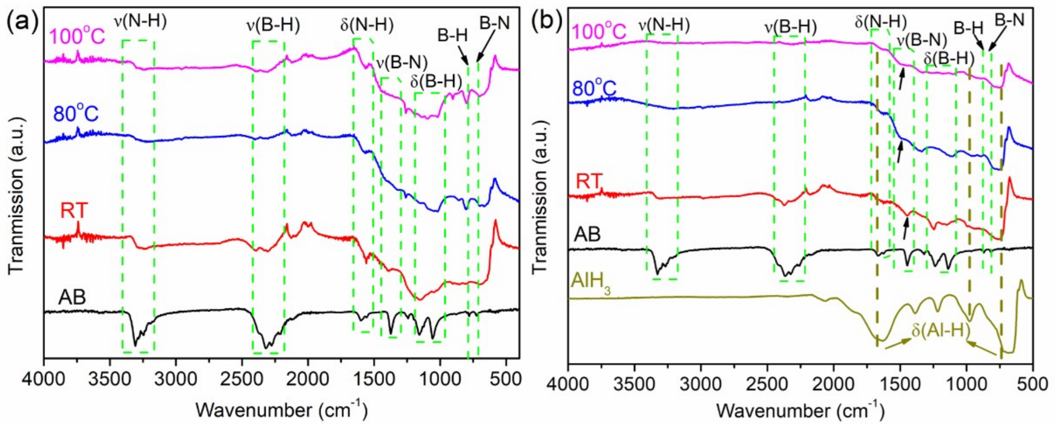

Figure S5. FT-IR patterns of (a) 30AB@CMK-5 and (b) (30AB:30AlH_3)@CMK-5 after the release of hydrogen at different temperatures.
